# Clinical course and surgical outcomes of cerebral cavernous malformations: Insights from a large single-center experience

**DOI:** 10.1016/j.bas.2026.107454

**Published:** 2026-09-22

**Authors:** Pavlina Lenga, Janis T. Linke, Edgar Tessmann, Bogdana Suchorska, Johannes Walter, Sandro M. Krieg, Moritz Scherer

**Affiliations:** a Department of Neurosurgery, Heidelberg University Hospital, Heidelberg, Germany; b Medical Faculty of Heidelberg, Heidelberg University, Heidelberg, Germany; c Institute of Medical Informatics, Heidelberg University Hospital, Heidelberg, Germany

**Keywords:** Cavernous malformation, Hemorrhage, Outcome, Postoperative complications, Surgery

## Abstract

**Introduction:**

Clinical decision-making for cerebral cavernous malformations (CCMs) requires balancing hemorrhage risk against surgery-related morbidity, yet clinically applicable risk stratification remains limited.

**Research question:**

To examine hemorrhage during recorded follow-up, postoperative complications, and functional outcomes after conservative and surgical management of CCMs.

**Material and methods:**

We retrospectively analyzed 268 patients treated between 2014 and 2021. Clinical, radiological, and treatment variables were collected. Functional outcome was assessed using the modified Rankin Scale (mRS), analyzed as an ordinal outcome using paired nonparametric tests. Recorded untreated observation was censored at surgery; hemorrhage rates were reported with exact 95% confidence intervals (CIs).

**Results:**

At baseline, most patients presented with hemorrhage and 25.4% of lesions were infratentorial. Most were managed conservatively (72.8%), whereas 27.2% underwent surgery. Median time from diagnosis to surgery was 48 days. Postoperative neurological complications occurred in 20/73 patients (27.4%), including seizure-related events (11.0%), diplopia in (4.1%), and hemiparesis in (4.1%). Among 68 surgical patients with paired assessments, median mRS was 1 [IQR 1–1] at baseline and 0 [0–1] at last follow-up; residual disability was greater in brainstem lesions. Two new radiographic hemorrhages were documented during 250.3 person-years in 129 patients with untreated observation (0.80/100 person-years; 95% CI 0.10–2.89). Age was not associated with postoperative complications (OR 1.01/year, 95% CI 0.97–1.04).

**Discussion and conclusion:**

In this tertiary-center cohort, favorable functional recovery was observed in selected surgical patients, but brainstem CCMs remained associated with residual morbidity. Sparse hemorrhage events and confounding by indication precluded treatment comparisons or a validated risk-stratification framework.

## Highlights

• Lesion location influence surgical and natural-history risk in cavernomas.
• Surgery enables favorable recovery in selected cavernoma patients.
• Brainstem cavernomas carry substantial operative morbidity.
• Risk-adapted management may improve cavernoma treatment decisions.

## 1 Introduction

Cerebrovascular malformations represent an important group of intracranial pathologies and carry a significant clinical and socioeconomic burden due to their variable natural and clinical course (Flemming et al., 2017; Flemming, 2017). Cavernous malformations (CCM) account for 10-25% of intracranial vascular malformations, with an overall incidence of 0.1 - 0.8% in the general population (Flemming et al., 2017; Caton et al., 2026; Labauge et al., 2007; Dulamea et al., 2024). A recent study reported an age-standardized incidence of 2.01/100.000 patients (Halmela et al., 2024). Presentation in the general population is mostly sporadic; however, familial incidence is up to 20% (Al-Shahi Salman et al., 2012; Haasdijk et al., 2012).

The natural course of CCMs is heterogeneous, ranging from asymptomatic in most cases to presentation with neurologic deficits with recurrent symptomatic hemorrhages (Caton et al., 2026; Halmela et al., 2024; Kondziolka et al., 1995). Localization is of particular clinical importance as it dictates symptoms, and lesions situated in the basal ganglia or the brainstem are associated with higher morbidity (Washington et al., 2010; Abla et al., 2011). The reported annual hemorrhage risk per lesion ranges from 1.7% to 2.5%, but in previously hemorrhaged CCMs it is up to 30% at 5 years (Gross et al., 2017; Flemming et al., 2012; Kearns et al., 2019; Horne et al., 2016). Hemorrhage rates depend on differences between patient cohorts, with prior hemorrhage and brainstem location accounting as the strongest predictors of future bleeding (Gross et al., 2017; Li et al., 2014).

Management of CCMs remains a subject of ongoing debate, with conservative or surgical therapy as the main strategies. At the same time, radiotherapy or medication is not a standard therapy and is only used subordinately (Akers et al., 2026; Lunsford et al., 2010; Hsu et al., 2007). Surgical resection provides a potential treatment, particularly in cases of recurrent hemorrhage (Akers et al., 2026; Rosenow et al., 2013; Nico et al., 2024; Al-Shalchy et al., 2025). Furthermore, surgical resection of CCMs has been shown to reduce seizure recurrence in patients with CCM-associated epilepsy (Rosenow et al., 2013; Nico et al., 2024; Baumann et al., 2007). However, surgery itself carries significant perioperative risks of neurological deficits, especially when CCMs are located in the brainstem (Tatagiba et al., 2023; Garrett et al., 2009). Conservative management avoids surgical morbidity but leaves patients at risk for future hemorrhage and progressive neurological deficits (Al-Shahi Salman et al., 2012). Moreover, conservative management of previously hemorrhaged CCM can significantly burden patients by revoking driving or machinery operation license for up to two years (Driver and V.L. Agency, 2023; Marx et al., 2019). Although recent studies emphasize the importance of individualized, risk-adapted decision-making, there is still no universally accepted model for categorizing patients into high- or low-risk groups (Dulamea et al., 2025; Rauschenbach et al., 2024).

For brainstem CCMs, proposed grading systems include the Lawton–Garcia and Essen/Dammann–Sure systems for estimating surgical outcome and the BSCM score developed by Yang, Bozinov and colleagues to support treatment selection (Yang et al., 2022; Garcia et al., 2015; Santos et al., 2021). The international Delphi consensus led by Dammann and the updated Alliance guideline emphasize clinical history, anatomical accessibility, surgical timing, and individualized decisions (Akers et al., 2026; Dammann et al., 2022).

Accordingly, we sought to examine hemorrhage during recorded follow-up and postoperative complications in a large single-center cohort of patients with CCMs and to describe functional trajectories after conservative and surgical management. Associations were considered exploratory, not evidence of comparative treatment effectiveness or a validated prediction model.

## 2 Methods

### 2.1 Study design

We designed a retrospective study of patients who presented to the Department of Neurosurgery at Heidelberg University Hospital between 2014 and 2021 with a diagnosis of cerebral cavernous malformation (CCM). The study complied with the Declaration of Helsinki. This study was approved by the local ethics committee under the number S-667/2021. Written informed consent was waived due to the study's retrospective nature. All patients underwent either conservative or surgical treatment after discussion of each case and consensus among the department's senior neurosurgeons, following guidelines and standardized operating procedures. Surgical resections were performed by senior neurosurgeons with experience in CCM surgery. The conservative management consisted of magnetic resonance imaging (MRI) and clinical follow-up at our outpatient clinic.

Selection considered the clinical presentation, hemorrhage, neurological deficits, lesion location and perceived accessibility, and senior neurosurgical judgment. The exact interval from the latest hemorrhage, brainstem midline crossing, lesion dimensions, and detailed operative anatomy were not systematically encoded. Therefore, their independent influence on selection could not be quantified, and no brainstem grading score was retrospectively reconstructed.

### 2.2 Data collection

Clinical data were retrieved from the institution's electronic medical records. The following data were collected: (1) age at index diagnosis/presentation, (2) sex, (3) symptoms at presentation as well as at the 1st, 2nd and 3rd recorded follow-up, (4) comorbidities, (5) modified Rankin Scale (mRS), (6) localization, (7) hemorrhagic presentation at index diagnosis, (8) multiplicity, (9) presence of developmental venous anomaly (DVA), (10) anticoagulant use at presentation, (11) treatment form, (12) occurrence of postoperative complications, (13) postoperative deficits, and (14) radiographic status at follow-up. The outcome assessment was performed using the modified Rankin Scale (mRS) at baseline, discharge after surgery, and follow-up.

The patient was the unit of analysis. In patients with multiple CCMs, multiplicity was recorded as a patient-level characteristic and localization referred to the index lesion recorded in the dataset; each patient contributed once. Anticoagulant exposure referred to use at index presentation, not a prescription initiated because of the CCM diagnosis. It did not include antiplatelet therapy. Specific agents, continuation, interruption, resumption, and new prescriptions during follow-up were not systematically available.

The mRS was retrospectively reconstructed from contemporaneous neurological examinations, functional descriptions, discharge summaries, and follow-up documentation. The last available valid recorded follow-up score, rather than the lowest score across follow-up, was selected. Follow-up visits were not fixed study time points; the number of available assessments is reported separately for each management group. A valid score could contribute to functional analysis even if its date could not support an interval calculation.

The variable previously labeled “previous bleeding” represented recorded hemorrhagic presentation at index diagnosis, not the number of earlier hemorrhages. Baseline hemorrhagic status was not uniformly adjudicated as symptomatic hemorrhage. A new radiographic hemorrhage during follow-up required an explicit report of new CCM bleeding, rather than stable blood products. Rebleeding denoted a subsequent hemorrhage in a patient with hemorrhagic presentation; a new hemorrhage without baseline hemorrhage was classified separately as a first recorded event. Symptomatic hemorrhage required new or worsened symptoms temporally attributable to the radiographic event. Event timing was based on the dated follow-up record documenting detection; exact onset was not systematically recorded.

Recorded untreated observation began at the recorded diagnosis date and ended at the first new radiographic hemorrhage, surgery, or the last documented clinical/radiological follow-up, whichever occurred first. Non-event observations were censored at surgery or last follow-up. Death was not a systematically captured endpoint. Patients without positive, chronologically useable intervals did not contribute person-time. Surgical dates inconsistent with admission, discharge, or postoperative follow-up chronology were excluded from surgery-dependent interval analyses; no dates were guessed. Postoperative functional follow-up was measured from surgery and conservative follow-up from diagnosis. Table 4 reports diagnosis-to-follow-up intervals in both groups to identify a common time origin for its mixed-management presentation.

Postoperative neurological complications comprised focal neurological deficits and seizure-related events recorded during the index surgical admission through discharge. They were analyzed separately from perioperative hemorrhagic/CSF-related events, residual-lesion progression, and revision surgery. The recorded principal category was used for the mutually exclusive Fig. 1 summary; a combined seizure/aphasia entry was assigned to the seizure-related group. Persistence was reported only when the same deficit remained documented at last follow-up; non-documentation did not establish resolution or permanence. Outcome ascertainment was limited to the institutional record and external reports incorporated into it, without systematic access to all outside records.

**Fig. 1:**
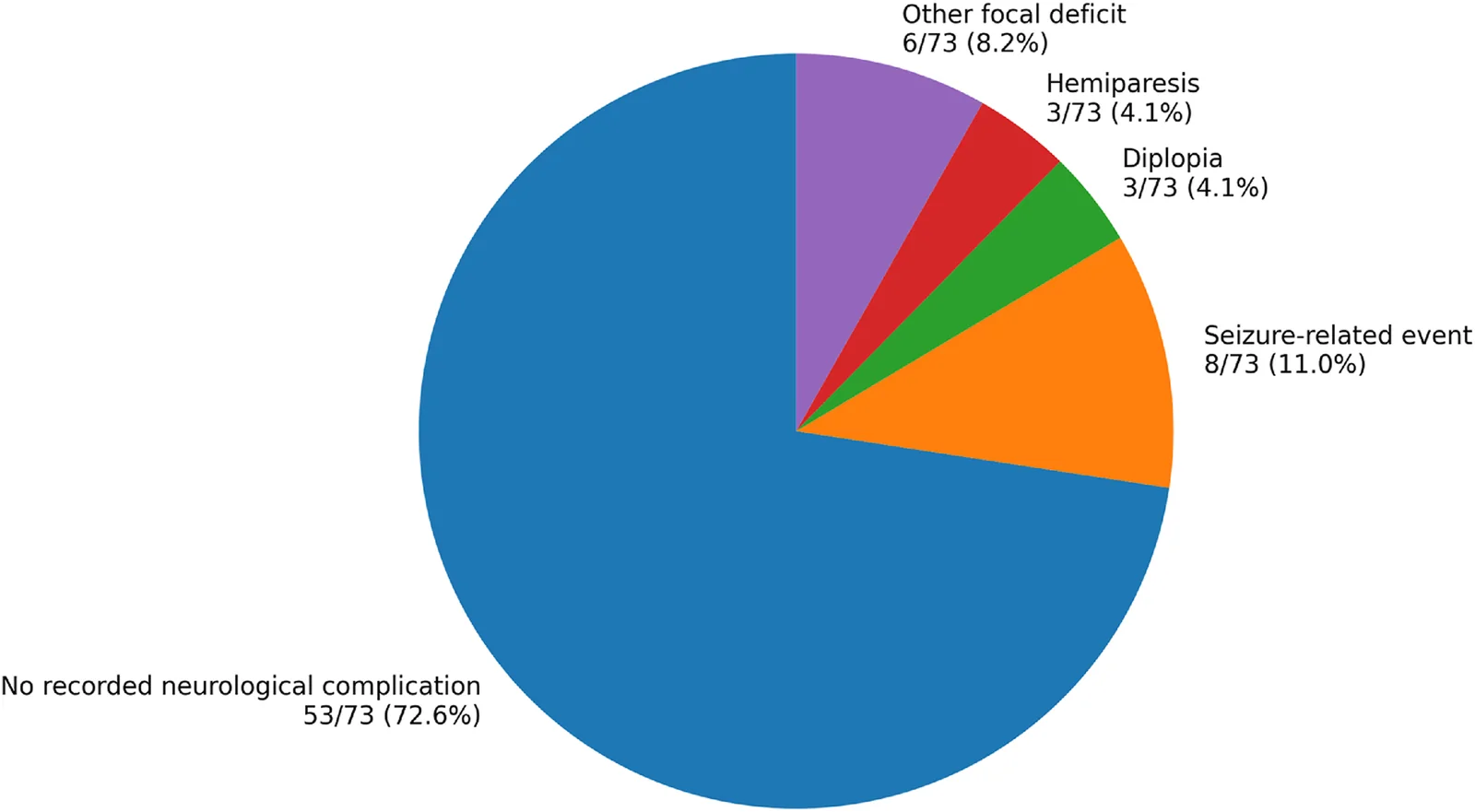
**Postoperative neurological complications after surgical resection** (n = 73). The mutually exclusive principal recorded categories are no neurological complication, 53/73 (72.6%); seizure-related event, 8/73 (11.0%); diplopia, 3/73 (4.1%); hemiparesis, 3/73 (4.1%); and other focal deficit, 6/73 (8.2%). One seizure-related event also included aphasia. These are index-admission events, not a permanent-deficit rate. Technical events and revisions are reported separately.

### 2.3 Statistical analysis

Continuous variables are summarized as mean ± standard deviation (SD), with follow-up intervals and ordinal mRS scores reported as median [interquartile range (IQR)]. Baseline group comparisons used Welch's *t*-test for age, the Mann–Whitney *U* test for mRS, and Fisher's exact test for categorical variables. These comparisons describe clinical selection rather than treatment effects. Postoperative neurological complications were analyzed only among the 73 surgical patients (20 events). Age was assessed by univariable logistic regression, with an unadjusted odds ratio (OR) per year and Wald 95% CI; categorical associations used Fisher's exact tests and exact conditional ORs with 95% CIs. No finite OR was reported for zero-cell or invariant exposures. No multivariable complication model or ROC-AUC was retained because the small number of events and sparse covariate strata did not support reliable adjusted risk prediction. Hemorrhage incidence was calculated per 100 person-years with exact Poisson 95% CIs. Only two subsequent hemorrhages were identified, precluding multivariable Cox regression. The mRS was treated as ordinal: repeated assessments in complete surgical pairs were evaluated using the Friedman test and pairwise Wilcoxon signed-rank tests, with Bonferroni correction for the three comparisons. Conservative baseline-to-follow-up change used a paired Wilcoxon signed-rank test. Wilcoxon tests used a two-sided normal approximation with tie correction, without continuity correction; zero differences were omitted. These methods do not assume normally distributed mRS values; they do not establish causality or equal clinical spacing between score categories. Localization-specific results were descriptive; McNemar's test and longitudinal ordinal regression were not retained. Missing outcomes were not imputed. Except for the three surgical paired comparisons, analyses were exploratory without multiplicity correction. Statistics for the revision were calculated using SciPy and statsmodels in Python, with matplotlib for figures. Two-sided p-values <0.05 were considered statistically significant.

## 3 Results

### 3.1 Patient data

Our cohort comprised 268 adult patients with cerebral cavernous malformations (CCMs) with a mean age of 44.4 ± 15.2 years, of whom 64.9% were women. The majority of the patients had hemorrhagic presentation (179/268, 66.8%). Motor and cranial nerve deficits at admission were uncommon (16/268, 6.0%, and 17/268, 6.3%, respectively) and were not mutually exclusive with hemorrhage. Most lesions were supratentorial (74.6%), 8.6% of patients had multiple CCMs, and 6.3% had an associated DVA. Anticoagulant use was documented at presentation in 7/268 patients (2.6%); all seven were managed conservatively. Most patients were managed conservatively (195/268, 72.8%). Surgical patients were younger (39.1 ± 14.8 versus 46.4 ± 14.9 years; p < 0.001) and more frequently had hemorrhagic presentation (68/73, 93.2%, versus 111/195, 56.9%; p < 0.001). A detailed presentation of patient characteristics is provided in Table 1.

**Table 1:** Demographic and baseline characteristics.

Characteristics	Overall	Conservative	Surgical	p-value
**Total patients (n)**	268	195	73	
Age at index diagnosis/presentation (mean ± SD, years)	44.4 ± 15.2	46.4 ± 14.9	39.1 ± 14.8	<0.001
Sex (n, %)				1.000
Male	94 (35.1)	68 (34.9)	26 (35.6)	
Female	174 (64.9)	127 (65.1)	47 (64.4)	
Hemorrhagic presentation at index diagnosis (n, %)	179 (66.8)	111 (56.9)	68 (93.2)	<0.001
Multiplicity of CCMs (n, %)	23 (8.6)	17 (8.7)	6 (8.2)	1.000
Developmental Venous Anomaly (DVA) (n, %)	17 (6.3)	13 (6.7)	4 (5.5)	1.000
Baseline anticoagulant use (n, %)	7 (2.6)	7 (3.6)	0 (0)	0.195
Location (n,%)				0.753
Infratentorial	68 (25.4)	51 (26.2)	17 (23.3)	
Supratentorial	200 (74.6)	144 (73.8)	56 (76.7)	
Neurological status on admission (n, %)				
Motor deficit (MD, %)	16 (6.0)	13 (6.7)	3 (4.1)	0.569
Cranial nerve deficits (CND, %)	17 (6.3)	8 (4.1)	9 (12.3)	0.022
mRS on admission (median [IQR]; range)	1 [0–1]; 0–3	1 [0–1]; 0–3	1 [1–1]; 0–3	0.002
Diagnosis to surgery (days, median [IQR])			48 [6–231] (n = 67)	
Last-follow-up duration (months, median [IQR])		23.0 [12.9–52.5] (n = 62)	13.1 [3.6–25.6] (n = 63)	
Valid last-follow-up mRS, n (%)	131 (48.9)	63 (32.3)	68 (93.2)	—
Valid mRS at recorded FU1/FU2/FU3, n	131/79/37	63/31/13	68/48/24	—
No valid follow-up mRS, n (%)	137 (51.1)	132 (67.7)	5 (6.8)	—
Useable follow-up ≥12 months, n	83	48	35	—

*Data* are n (%) unless stated otherwise. SD: standard deviation; IQR: 25th–75th percentile; *mRS: modified Rankin Scale; FU: recorded follow-up. Baseline p-values compare the conservative and surgical groups using Welch's t-test for age, Mann–Whitney U for mRS, and Fisher's exact test for categorical variables. Follow-up duration is diagnosis-to-last-follow-up for conservative care and surgery-to-last-follow-up for surgery; these different time origins are not directly compared. Six inconsistent operative chronologies were excluded from surgery-dependent timing, and one conservative date preceded diagnosis. Otherwise valid clinical scores were retained. Follow-up visits were not fixed scheduled study time points.*

Valid mRS values at the first, second, and third recorded follow-ups were available in 68, 48, and 24 surgical patients and 63, 31, and 13 conservatively managed patients, respectively. Five surgical patients and 132 conservative patients had no valid follow-up mRS. Among patients with useable chronology, surgery-to-last-follow-up duration was 13.1 months [IQR 3.6–25.6; n = 63], and diagnosis-to-last-follow-up duration was 23.0 months [12.9–52.5; n = 62] in the conservative cohort. Follow-up of at least 12 months was documented in 35 surgical and 48 conservative patients. The diagnosis-to-surgery interval was 48 days [IQR 6–231; n = 67]. Six inconsistent surgical chronologies were excluded from surgery-dependent interval calculations, and one conservative follow-up date preceded diagnosis. These exclusions did not remove otherwise valid clinical scores.

### 3.2 Postoperative complications

Among surgically treated patients, 53/73 (72.6%) had no recorded postoperative neurological complication. The 20/73 patients (27.4%) with such complications included seizure-related events in 8/73 (11.0%), diplopia in 3/73 (4.1%), and hemiparesis in 3/73 (4.1%) (Fig. 1). One seizure-related event also included aphasia. Other recorded focal deficits occurred in 6/73 patients (8.2%): one each of hemianopsia, hand paresis, dysphagia, hemihypaesthesia, isolated aphasia, and gait disturbance. Seizure-related events do not imply new-onset epilepsy. The same focal deficit remained explicitly documented at last follow-up in 5/73 patients (6.8%); lack of a subsequent symptom entry was not interpreted as definitive resolution. Two perioperative hemorrhagic/CSF-related events were recorded separately: hemorrhage with CSF fistula (n = 1) and epidural hemorrhage with hydrocephalus (n = 1). These categories may overlap with neurological complications and must not be summed as independent patient counts.

### 3.3 Exploratory risk assessments

#### 3.3.1 Analysis of postoperative complication associations

In the revised univariable analysis restricted to the surgical cohort, age was not associated with postoperative neurological complications (OR 1.01 per year, 95% CI 0.97–1.04; p = 0.606). Events occurred in 8/17 patients with infratentorial lesions and 12/56 with supratentorial lesions (exact conditional OR 3.20, 95% CI 0.87–11.82; Fisher's exact p = 0.060). Multiplicity and DVA showed imprecise associations (Table 2). All 20 events occurred among the 68 patients with hemorrhagic presentation, compared with 0/5 without hemorrhagic presentation; no finite OR or protective interpretation was reported. No surgical patient received baseline anticoagulation, so that exposure could not be evaluated. These unadjusted analyses do not establish independent predictors or validated discrimination.

**Table 2:** Exploratory unadjusted associations with postoperative neurological complications.

Variable	Events/patients	Unadjusted OR (95% CI)	p-value
Age, per year	73 patients; 20 events	1.01 (0.97–1.04)	0.606
Hemorrhagic presentation, yes vs no	20/68 vs 0/5	Not finite^a^	0.314
Infratentorial vs supratentorial	8/17 vs 12/56	3.20 (0.87–11.82)	0.060
Multiple vs solitary CCM	2/6 vs 18/67	1.36 (0.11–10.42)	0.663
DVA present vs absent	2/4 vs 18/69	2.79 (0.19–41.09)	0.301
Baseline anticoagulant use, yes vs no	0/0 vs 20/73	Not estimable^b^	—

The analysis includes all 73 surgical patients and 20 patients with recorded postoperative neurological complications; there were no missing values for the listed covariates. Age: univariable logistic regression with a Wald CI and p-value. Categorical variables: exact conditional OR with an exact 95% CI and Fisher's exact two-sided p-value. No adjustment for other covariates or multiplicity was performed.

a All 20 events occurred after hemorrhagic presentation, with zero events among five patients without it; no finite OR or protective interpretation is reported.

b No surgical patient had baseline anticoagulant exposure, so the association is not estimable. These are not independent predictors or a validated risk model.

#### 3.3.2 Hemorrhage during recorded untreated observation

After excluding missing or chronologically inconsistent intervals, 129 patients contributed 250.3 person-years of recorded untreated observation; 67 surgical patients contributed time only until surgery. Median observation time was 11.2 months [IQR 1.1–25.6] and mean observation time was 23.3 months. Two new radiographic hemorrhages were documented, both in conservatively managed patients, yielding 0.80 events per 100 person-years (exact 95% CI 0.10–2.89). One was a first recorded, asymptomatic hemorrhage in a patient without hemorrhagic presentation, detected at 11.3 months; the other was a symptomatic rebleed after hemorrhagic presentation, detected at 60.2 months. The conservative-only estimate was 1.02 events per 100 person-years (95% CI 0.12–3.67; 62 patients, 197.0 person-years). Table 3 separates these outcomes and denominators. No multivariable rebleeding model was retained because one recurrent event cannot support stable adjusted estimation. No follow-up entry was coded as recurrent CCM hemorrhage after surgery; this is a documentation finding, not proof of complete protection after resection.

**Table 3:** New radiographic hemorrhage during recorded untreated observation.

Observation/endpoint	Patients, n	Events, n	Person-years	Rate/100 person-years (95% CI)
All evaluable untreated observation	129	2	250.3	0.80 (0.10–2.89)
Conservative management only	62	2	197.0	1.02 (0.12–3.67)
Before surgery only	67	0	53.3	0.00 (0.00–6.92)
Hemorrhagic presentation: rebleeding	94	1	160.9	0.62 (0.02–3.46)
No hemorrhagic presentation: first recorded hemorrhage	35	1	89.4	1.12 (0.03–6.23)

Rates use exact Poisson 95% CIs. Observation begins at the recorded diagnosis and ends at the first documented new hemorrhage, surgery, or last available follow-up. Six inconsistent surgical chronologies and conservative records without a useable positive interval were excluded. One new hemorrhage was a first, asymptomatic event without hemorrhagic presentation; the other was a symptomatic rebleed after hemorrhagic presentation. Detection dates, rather than independently adjudicated onset dates, were used. Management and presentation subgroups overlap and must not be added. These retrospective diagnosis-based intervals may precede referral and are not a population-based natural-history estimate. No Cox model is reported.

### 3.4 Clinical outcome by lesion location in both management groups

Patients with supratentorial CCMs, particularly in the frontal and temporal lobes, presented with minimal symptoms and retained low mRS scores at the last recorded follow-up. By contrast, lesions in deep locations, such as the basal ganglia and brainstem, showed greater residual functional burden (median last-follow-up mRS 1 [IQR 0.5–1.5] and 1 (Flemming et al., 2017), respectively, versus 0 [0–0] for frontal lesions; Table 4). Table 4 includes both management groups, identifies their composition at each location, and reports diagnosis-to-last-follow-up duration, available denominators, baseline hemorrhagic status, and new hemorrhage events. All recorded locations are included, including corpus callosum with no evaluable follow-up. Among the 11 patients with last-follow-up mRS ≥2, 10 had hemorrhagic presentation and one had a documented subsequent symptomatic rebleed. Location-specific findings are descriptive and are not treatment-effect comparisons.

### 3.5 Clinical outcome in patients with surgical treatment

Patients who underwent surgical intervention showed an overall improvement in recorded functional status. Among 68 patients with baseline, discharge, and last-follow-up mRS, the median score was 1 [IQR 1–1], 1 [0–1], and 0 [0–1], respectively (Friedman χ^2^ = 41.65, p < 0.001). Fig. 2(A) shows the distribution in these same 68 patients at each assessment. Paired Wilcoxon comparisons were not significant between baseline and discharge (Bonferroni-adjusted p = 0.867), but scores were lower at last follow-up than at baseline and discharge (both adjusted p < 0.001). These within-group changes may reflect recovery from the presenting event, selection, and treatment; they do not demonstrate a causal benefit of surgery.

**Fig. 2:**
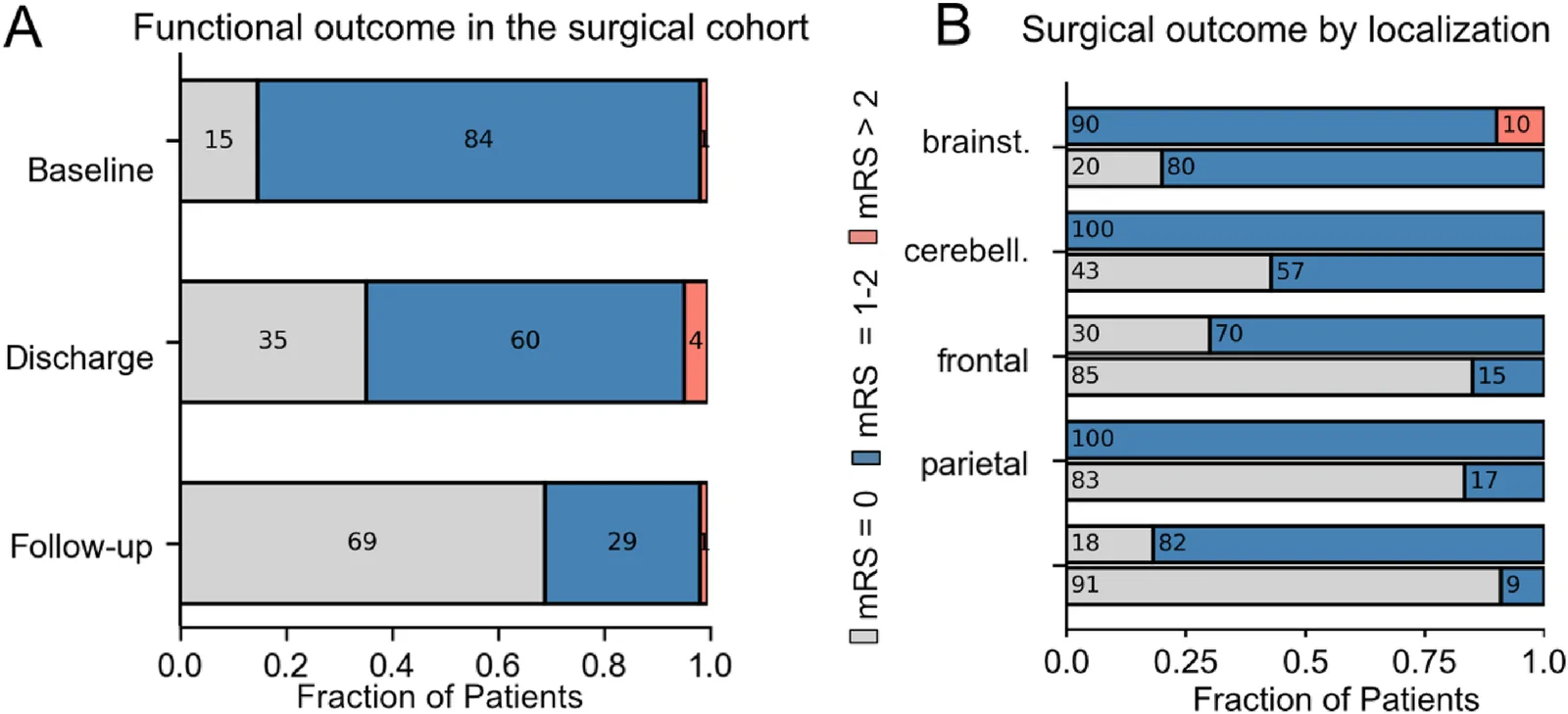
**Clinical outcome of patients with surgical intervention by CCM localization**. *(A) Distribution of mRS at baseline, discharge, and last recorded follow-up in the same 68 patients with complete assessments. Global change was assessed using the Friedman test (χ*^*2*^*=41.65, p<0.001); paired Wilcoxon comparisons with Bonferroni correction were non-significant for baseline versus discharge (p=0.867) and significant for each comparison with last follow-up (both p<0.001). (B) Paired baseline and last-follow-up distributions for the original five anatomical groups: brainstem (n=10), cerebellum (n=7), frontal (n=20), parietal (n=12), and temporal (n=11). Other recorded locations are reported in*Table 4*. Categories are mRS 0, 1–2, and >2. No localization-specific hypothesis tests or causal treatment-effect claims are attached to panel B. Last follow-up is used rather than the lowest observed score.*

In patients with conservative treatment and available paired assessments (n = 63), median mRS was 1 [IQR 0–1] at baseline and 0 [0–1] at last follow-up (paired Wilcoxon p = 0.034). Scores decreased in 18 patients, were unchanged in 39, and increased in six. The low follow-up availability in this group precludes a reliable comparison with the surgical cohort.

Consistent with the overall cohort (Table 4), lobar localizations showed favorable recorded functional outcomes, whereas brainstem CCMs retained greater disability. Fig. 2(B) depicts paired baseline and last-follow-up distributions for the five locations shown in the original figure, without location-specific significance claims. The cohort included 40 brainstem lesions; 10 were treated surgically and all 10 had hemorrhagic presentation. Postoperative neurological complications occurred in 5/10 brainstem cases versus 15/63 non-brainstem surgical cases (Fisher's exact p = 0.124). Median last-follow-up mRS in the brainstem surgical subgroup was 1 [IQR 1–1.75]. These small subgroup results are exploratory.

**Table 4:** Paired mRS outcomes and follow-up by all recorded anatomical locations, including conservative and surgical management.

Location	Cohort n	Paired FU n (S/C)	Baseline mRS, median [IQR]	Last mRS, median [IQR]	Diagnosis-to-FU months, median [IQR]	Baseline hemorrhage, n^a^	New hemorrhage, n^a^
Frontal	78	39 (20/19)	1 [0–1]	0 [0–0]	21.6 [12.7–42.5] (n = 39)	28	1
Temporal	47	25 (11/14)	1 [0–1]	0 [0–0]	24.3 [15.6–60.2] (n = 25)	20	1
Brainstem	40	21 (10/11)	1 [1–1]	1 [1–1]	15.6 [11.2–36.3] (n = 21)	15	0
Parietal	29	18 (12/6)	1 [1–1]	0 [0–0]	21.9 [13.8–38.2] (n = 17)	12	0
Cerebellum	28	9 (7/2)	1 [1–1]	1 [0–1]	4.1 [3.2–20.0] (n = 9)	7	0
Basal ganglia	17	7 (1/6)	1 [1–1.5]	1 [0.50–1.50]	44.0 [18.8–50.1] (n = 7)	5	0
Occipital	11	5 (4/1)	1 [0–1]	1 [0–1]	11.3 [7.1–12.9] (n = 5)	5	0
Diencephalon	6	1 (0/1)	1 [1–1]	1 [1–1]	3.5 [3.5–3.5] (n = 1)	0	0
Insula	5	2 (1/1)	1 [1–1]	1.50 [0.75–2.25]	53.1 [35.3–70.9] (n = 2)	1	0
Limbic	3	1 (0/1)	1 [1–1]	1 [1–1]	37.4 [37.4–37.4] (n = 1)	1	0
Intraorbital	3	3 (2/1)	1 [1–1]	0 [0–0]	7.5 [5.5–9.6] (n = 3)	1	0
Corpus callosum	1	0 (0/0)	—	—	—	0	0

S/C: surgical/conservative patients contributing valid paired mRS assessments; FU: last recorded follow-up; IQR: 25th–75th percentile.

a Counts are among patients contributing paired mRS. New hemorrhage includes one first event and one symptomatic rebleed. Diagnosis-to-follow-up uses a common origin for both management groups, excludes one chronologically invalid conservative date, and is not the postoperative interval. All 12 recorded locations are retained; no minimum subgroup size was applied. Corpus callosum has no evaluable follow-up. The location label “intraorbital” is retained as recorded without pathological reclassification. Analyses are descriptive.

Functional improvement was not immediate: among the 68 surgical patients with paired assessments, 18/68 (26.5%) had a lower mRS at discharge, 39/68 (57.4%) were unchanged, and 11/68 (16.2%) had a higher score. By last follow-up, 42/68 (61.8%) had a lower score than at baseline, 20/68 (29.4%) were unchanged, and 6/68 (8.8%) remained worse. These patterns are also reflected in Fig. 2(A).

Among surgically treated patients, revision surgery was required in three cases (3/73, 4.1%): one each for wound infection, cerebrospinal fluid leak, and resection of a residual lesion following progression. Residual-lesion progression was not classified as rebleeding. Extent of resection and a standardized postoperative imaging assessment were not systematically available, so a complete-resection rate could not be established.

## 4 Discussion

### 4.1 Principal findings

This study describes the clinical course and surgical outcomes of CCMs within a tertiary referral cohort. First, patients selected for surgery differed substantially from those managed conservatively, particularly in age and hemorrhagic presentation. Second, postoperative neurological complications were documented in 20/73 surgical patients, but unadjusted associations were imprecise and did not establish independent predictors. Third, functional recovery after surgery was generally favorable in the available paired observations, with greater residual morbidity in brainstem CCMs. Only two new radiographic hemorrhages were documented during recorded untreated observation, of which one was a symptomatic rebleed. These findings do not establish a practical risk-stratification model.

### 4.2 Natural history and rebleeding risk

Our cohort of 268 patients has demographic and clinical features comparable to previously described CCM populations, although referral selection and incomplete follow-up limit representativeness. The mean age of 44 years is consistent with prior reports of symptomatic presentation in middle adulthood (Dulamea et al., 2024; Akers et al., 2026; El-Koussy et al., 2011). Although 64.9% of our cohort were female, larger studies suggest equal sex distribution but report similar patterns (Flemming, 2017; Dulamea et al., 2024; Gross et al., 2017). Lesion distribution was comparable to that reported in published series, with 75% supratentorial localization (Dulamea et al., 2024; Gross et al., 2017). Established CCM natural-history studies identify hemorrhagic presentation and brainstem or deep location as important risk factors (Al-Shahi Salman et al., 2012; Gross et al., 2017; Flemming et al., 2012; Li et al., 2014; Taslimi et al., 2016), and temporal clustering can influence observed rates (Barker et al., 2001). However, our two subsequent hemorrhages—only one of which was a rebleed—cannot independently confirm these associations or support multivariable modeling. Sex has not consistently been a predictor in previous cohorts (Gross et al., 2017; Flemming et al., 2012, 2023); no sex-specific conclusion can be drawn here. The low recorded rate should not be interpreted as a definitive natural-history estimate because observation was retrospective, some diagnoses preceded referral, and follow-up was incomplete.

### 4.3 Surgical outcomes and interpretation

Surgical treatment was associated with lower recorded mRS scores at follow-up, although this observation does not establish causality. The fact that improvement was not always immediate but continued until follow-up is clinically relevant. Early postoperative deficits, especially following deep or brainstem surgery, should not automatically be interpreted as indicative of poor long-term outcomes (Abla et al., 2011; Al-Shalchy et al., 2025; Velz et al., 2021; Hori et al., 2023; Albalkhi et al., 2023). Recovery was more favorable for lobar lesions and less favorable for brainstem CCMs, consistent with previous reports (Yang et al., 2022). Nevertheless, the composite complication rate includes seizure-related events as well as focal deficits, and it is not a rate of permanent surgical disability. The revised univariable age estimate was close to unity, so this cohort does not establish age-related operative risk. Infratentorial complications were numerically more frequent, but the exact CI was wide and included unity. No inference of protection from baseline hemorrhage is justified: only five surgical patients lacked hemorrhagic presentation, and none had a recorded complication. Timing and operative anatomy are recognized considerations (Qian et al., 2025; Catapano et al., 2023), but lesion-specific surgical corridors, cleavage planes, and hemorrhage-to-surgery timing were not systematically captured and cannot explain an association in this dataset.

The clinical question remains the balance between natural history and operative risk. Surgical intervention may be considered when the lesion is accessible and the untreated risk is elevated, such as after symptomatic hemorrhage or in cases of CCM-related epilepsy (Akers et al., 2026; Rosenow et al., 2013; Nico et al., 2024). Conversely, the threshold for intervention should be higher for deep-brainstem lesions, where both the natural history and the surgical procedure are associated with substantial morbidity (Akers et al., 2026; Tatagiba et al., 2023; Garrett et al., 2009). Our records did not contain a postoperative CCM rebleed, but complete resection was not systematically verified; therefore, the cohort cannot establish durable protection specifically attributable to complete resection. One revision was performed for residual-lesion progression, which is distinct from hemorrhage.

### 4.4 Clinical implications

Overall, our data illustrate the need to consider both untreated disease burden and operative morbidity rather than a binary surgery-versus-observation approach. Anatomical location remains an important consideration in management strategy (Akers et al., 2026; Taslimi et al., 2016). For accessible lesions, particularly following symptomatic hemorrhage or in cases of epilepsy, resection may be considered in the context of guideline recommendations and individual circumstances (Akers et al., 2026; Rosenow et al., 2013; Gao et al., 2020). In contrast, brainstem CCMs represent a challenging subgroup because of neurological disease burden and operative risk. These cases require highly selective indications, careful timing, and management in experienced centers (Akers et al., 2026; Tatagiba et al., 2023; Garrett et al., 2009). The primary value of this study is the description of clinical selection, postoperative events, and functional trajectories in routine care. It does not demonstrate that a new algorithm improves patient selection or validate a bedside risk calculator.

The Lawton–Garcia system incorporates lesion size, axial-midpoint crossing, DVA, age, and hemorrhage-to-surgery interval; the Essen/Dammann–Sure classification is another outcome-oriented tool (Garcia et al., 2015; Santos et al., 2021). The BSCM score instead models treatment-selection considerations (Yang et al., 2022). These instruments and the Dammann Delphi consensus address different aspects of decision-making and do not substitute for detailed case assessment (Dammann et al., 2022). With only 10 surgical brainstem cases and incomplete score variables, our cohort neither validates these systems nor supplies an alternative. The revised interpretation is therefore consistent with the updated Alliance guideline (Akers et al., 2026), without proposing a new decision algorithm.

### 4.5 Limitations

We acknowledge several limitations of our study. First, case inclusion was retrospective, based on referral to a tertiary academic center, which may have introduced bias typically associated with similar study protocols. Secondly, treatment allocation was nonrandomized, introducing confounding by indication. While treatment decisions were made by senior consultants of vascular neurosurgery or a multidisciplinary board, surgeons may have selected healthier patients with more accessible lesions, while higher-risk cases were managed conservatively (Davies et al., 2017). Baseline imbalances and natural recovery after a presenting hemorrhage prevent attribution of within-group mRS change to surgery alone. Third, follow-up duration was heterogeneous and ascertainment differed substantially: last-follow-up mRS was available for 68/73 surgical patients but only 63/195 conservative patients. Events treated elsewhere could be missed unless external reports reached our institution. Recorded diagnosis dates sometimes preceded referral; the resulting diagnosis-based intervals are not equivalent to prospectively monitored natural history. Chronologically inconsistent dates were excluded from affected interval calculations without assigning replacement dates. Fourth, only two subsequent radiographic hemorrhages and 20 postoperative neurological complications were identified. The sparse event counts, five nonhemorrhagic surgical cases, small DVA and multiplicity strata, and lack of validation prevent robust multivariable prediction. The small number of patients with baseline anticoagulant exposure also limits conclusions; antiplatelet exposure and longitudinal medication changes were not captured, although previous reports address antithrombotic safety (Guranda et al., 2025; Bervini et al., 2019). Fifth, mRS was reconstructed from routine records and follow-up symptom documentation was not standardized; missing values were not imputed and symptom absence was not equated with resolution. Lesion dimensions, eloquence/depth, brainstem sublocation, midline crossing, detailed prior hemorrhage history, operative approach, extent of resection, and standardized postoperative imaging were not consistently available. As a tertiary referral cohort, this series is likely enriched for symptomatic and surgically relevant lesions, which may account for the high rate of hemorrhagic presentation and may limit generalizability to incidental CCMs.

### 4.6 Future directions

This study's limitations identify priorities for future risk-stratification and management research. Multicenter prospective studies with standardized imaging, outcome definitions, and follow-up are essential for developing and validating risk models. Collaborative studies will enable larger, more heterogeneous patient samples and yield more robust, generalizable risk estimates. Incorporating detailed imaging features may further enhance the prediction of future hemorrhage. High-resolution MRI sequences can more accurately quantify lesional permeability, assess surrounding hemosiderin, and detect associated DVAs (Caton et al., 2026; Tan et al., 2016). Genetic information, such as CCM gene mutations in familial cases, and circulating biomarkers could also further refine individualized risk estimates (Haasdijk et al., 2012; Fontes-Dantas et al., 2026). After appropriate validation, including these variables in digital risk calculators could enable clinicians to estimate hemorrhage and operative risks in real time, thereby supporting patient-specific decision-making. The present study does not determine the optimal timing of intervention. Cohort studies or decision-analysis models could evaluate whether risk-guided strategies improve outcomes, including patient-reported outcomes and quality-of-life measures (Akers et al., 2026). The effectiveness of emerging therapies, such as stereotactic radiotherapy or medical treatments, should also be explored in defined risk scenarios (Akers et al., 2026; Lunsford et al., 2010; Hsu et al., 2007). In summary, progress in CCM management will require collaborative, data-driven approaches, personalized risk assessment tools, and prospectively validated predictive models that are practical and applicable in clinical settings.

## 5 Conclusion

In this tertiary-center cohort, patients selected for surgery frequently had hemorrhagic presentation, and favorable functional outcomes were observed in patients with available follow-up, particularly in lobar lesions. Brainstem CCMs retained greater residual functional burden. However, sparse hemorrhage events, incomplete and differential follow-up, and confounding by indication preclude independent risk prediction or causal conclusions about management strategies. The study provides exploratory observations for counseling and prospective investigation, not a validated patient-selection framework.

## Ethics declaration

Written informed consent to take part in the study and to publish the article has been obtained from all participants or their legal representatives. The privacy rights of participants have been observed.

This study was performed in compliance with relevant laws, regulatory frameworks and guidelines where the research took place. This study was approved by the Ethics comitte Heidelberg. (Approval No. S-667/2021)

## Statement of authorship

All authors meet the criteria for authorship and have made substantial contributions to the conception, design, data acquisition, analysis, and interpretation of the study. All authors contributed to drafting or revising the manuscript and approved the final version.

## Ethical adherence

This study was conducted in accordance with the Declaration of Helsinki and approved by the local ethics committee (S-667/2021). The requirement for informed consent was waived due to the retrospective nature of the study.

## Financial disclosure

The authors state that there are no financial disclosures and no external funding was received for this study.

## Declaration of competing interest

The authors declare that they have no known competing financial interests or personal relationships that could have appeared to influence the work reported in this paper.
